# Case Report: Fully transvaginal extraperitoneal pelvic lymphadenectomy via retropubic access combined with radical vaginal hysterectomy or trachelectomy for early-stage cervical cancer

**DOI:** 10.3389/fsurg.2026.1775199

**Published:** 2026-03-13

**Authors:** Xiaobin Huang, Cankun Zhou, Yuyi Ou

**Affiliations:** Department of Gynaecology, Foshan Women and Children Hospital Affiliated to Guangdong Medical University, Foshan, China

**Keywords:** cervical cancer, extraperitoneal pelvic lymphadenectomy, radical vaginal hysterectomy, radical vaginal trachelectomy, sentinel lymph node, transvaginal natural orifice transluminal endoscopic surgery (vNOTES)

## Abstract

**Background:**

Scarless approaches are increasingly explored in the surgical management of early-stage cervical cancer. Transvaginal natural orifice transluminal endoscopic surgery (vNOTES) has been described for retroperitoneal access and, more recently, for radical hysterectomy and sentinel node biopsy. Here we report a fully transvaginal radical strategy that combines a distinct retropubic extraperitoneal corridor for vaginal endoscopic extraperitoneal lymphadenectomy (VEEL) with radical vaginal hysterectomy or trachelectomy.

**Case description:**

Five consecutive women (30–55 years) with FIGO 2018 stage IA2–IB1 cervical cancer underwent retropubic extraperitoneal VEEL followed by radical vaginal hysterectomy or radical vaginal trachelectomy. No abdominal trocar was used. Pelvic lymph node yields were 26, 21, 14, 34 and 10, respectively. At last follow-up (6–64 months), all patients were alive without evidence of disease. The most frequent postoperative morbidity was pelvic lymphocele/collections (2/5), including one symptomatic lymphocele requiring laparoscopic fenestration (Clavien–Dindo IIIb).

**Conclusion:**

Retropubic extraperitoneal VEEL combined with radical vaginal surgery may enable oncologically adequate nodal staging without abdominal scars. However, lymphocele/collections observed in this small series underscore the importance of careful patient selection, standardized lymphocele-prevention measures, and consideration of sentinel lymph node mapping to reduce lymphatic morbidity in appropriately staged early disease.

## 1 Introduction

Radical hysterectomy or radical trachelectomy with pelvic lymph node assessment remains a standard option for selected patients with early-stage cervical cancer, particularly when definitive chemoradiation is undesirable or fertility preservation is considered (1). In parallel, surgeons continue to refine techniques that aim to reduce surgical trauma and improve recovery while maintaining oncologic principles.

Transvaginal natural orifice transluminal endoscopic surgery (vNOTES) offers a scarless route and is increasingly used in gynecologic surgery (2). Retroperitoneal vNOTES has been reported for sentinel lymph node biopsy and pelvic staging (3–5), and vNOTES radical hysterectomy has been described by Baekelandt and colleagues (6, 7). Importantly, radical vaginal surgery (Schauta-type radical vaginal hysterectomy or trachelectomy) has a long historical precedent and can be integrated with minimally invasive nodal assessment.

Extraperitoneal lymphadenectomy avoids bowel manipulation and may reduce intraperitoneal contamination, adhesions, and postoperative ileus. Nonetheless, classic extraperitoneal nodal approaches may be associated with a higher risk of lymphocele compared with transperitoneal routes because lymphatic fluid is less readily absorbed by the peritoneum. Therefore, lymphatic-morbidity prevention and modern de-escalation strategies such as sentinel lymph node mapping warrant explicit consideration in early-stage disease, consistent with contemporary guidelines (1).

In this case series, we describe five consecutive women with FIGO 2018 stage IA2–IB1 cervical cancer treated using a retropubic extraperitoneal corridor created behind the descending pubic ramus to perform VEEL without intentional peritoneal entry, followed by radical vaginal hysterectomy or radical vaginal trachelectomy. We clarify how this retropubic access differs from previously published paracervical retroperitoneal vNOTES descriptions and discuss safety considerations, including the role of sentinel lymph node mapping.

## 2 Case description

Between January 2019 and July 2024, five consecutive Han Chinese women with FIGO 2018 stage IA2–IB1 cervical cancer underwent a fully transvaginal radical procedure at the Department of Gynecology, Foshan Women and Children Hospital (Affiliated to Guangdong Medical University). This case series is reported in accordance with the CARE reporting guideline (8).

Patients' ages ranged from 30 to 55 years (mean 39.8). Presenting features included abnormal cervical screening results, postcoital bleeding, persistent high-risk HPV infection, or increased vaginal discharge. Pelvic examination showed cervical ectropion/contact bleeding or post-conisation changes without parametrial induration or palpable lymphadenopathy. Table 1 summarises baseline case information.

**Table 1:** Summary of baseline case characteristics.

Patient	Age (years)	Presentation/exam/MRI	HPV & SCC-Ag	Preoperative pathology/FIGO 2018 stage
1	43	No abnormality; Grade II columnar epithelial ectopia of the cervix with contact bleeding; MRI: Endocervical thickening	HPV 18; SCC-Ag 0.85 ng/mL	Cervical adenocarcinoma at point 6/12 of the cervix; IB1
2	36	Contact vaginal bleeding for 3 months; No abnormality; MRI: no suspicious findings	HPV 16; SCC-Ag 1.56 ng/mL	HSIL/CIN3 with gland involvement; invasion suspected; VaIN3; suspected IB1
3	30	Persistent HPV infection for 5 years; Postoperative changes after cervical cold knife conization; MRI: no suspicious findings	HPV 16/52; SCC-Ag 0.60 ng/mL	Post-cone microinvasive SCC (width ∼5 mm, depth ∼4 mm); IA2
4	55	Cervical lesions were discovered 1 month ago; Mild cervical erosion-like and post-biopsy appearance of the cervix; MRI: Abnormal signal in the anterior lip of the cervix	Other high-risk HPV (+); SCC-Ag 1.50 ng/mL	HSIL/CIN3 fragments on biopsy/ECC; invasion suspected; IB1
5	35	Cervical lesions were discovered 2 month ago; Grade II cervical columnar epithelial ectopia; MRI: Multiple Nabothian cysts of the cervix	HPV 16; SCC-Ag 1.20 ng/mL	HSIL/CIN2–3 with suspected microinvasion; IA2

MRI, magnetic resonance imaging; SCC-Ag, squamous cell carcinoma antigen; HPV, human papillomavirus; CIN, cervical intraepithelial neoplasia.

All cases were reviewed at a multidisciplinary tumour board. Surgical options and the investigational nature of the fully transvaginal approach were discussed. Each patient provided written informed consent for the procedure and publication of de-identified clinical data and images. Patients with bulky or markedly endophytic tumours were not considered candidates.

## 3 Timeline

A consolidated timeline of the episode of care is provided in Table 2.

**Table 2:** Timeline of care for the five cases.

Time point	Key events and findings
Preoperative (weeks–months)	Abnormal screening and/or symptoms → HPV genotyping and cytology → colposcopy-directed biopsy confirming invasive cervical carcinoma; pelvic MRI showing localized disease without parametrial invasion or suspicious lymph nodes; FIGO 2018 staging IA2–IB1; multidisciplinary tumour board review and counselling.
Day 0 (surgery)	Early tumour containment with a circumferential vaginal sleeve/cuff; creation of a retropubic extraperitoneal working cavity (CO2 11 mmHg) and landmark-guided VEEL; completion of RVH or fertility-sparing RVT; closure of vaginal cuff or cervical stump; ureteral catheters removed at end of surgery.
Postoperative days 1–2	Routine recovery; bowel function returned by postoperative day 2 in all patients; monitoring for lymphatic leakage/lymphocele by symptoms and imaging as indicated.
Discharge	Hospital stay 10–27 days (mean 14.4). Symptomatic lymphocele was managed conservatively in three patients; one required re-intervention.
Follow-up (6–64 months)	Regular pelvic examination, cytology, SCC-Ag and cross-sectional imaging as clinically indicated; adjuvant therapy delivered to two patients based on final pathology; no recurrence observed at last follow-up.

## 4 Diagnostic assessment

Diagnostic assessment included cervical cytology (ThinPrep), high-risk HPV genotyping and colposcopy-directed biopsy in all patients. Histopathology confirmed invasive carcinoma (four squamous cell carcinomas and one adenocarcinoma) with associated CIN II–III in several cases. Pelvic MRI demonstrated localised cervical lesions without parametrial invasion or suspicious lymphadenopathy. Preoperative staging was FIGO 2018 IA2–IB1 (Table 1).

## 5 Therapeutic intervention

All procedures were performed under general anaesthesia in the lithotomy position by the Department of Gynecologic Oncology at Foshan Women and Children Hospital Affiliated to Guangdong Medical University. A consistent surgical team was led by a senior gynaecologic oncologist experienced in radical vaginal surgery and advanced laparoscopy.

**Figure 1:**
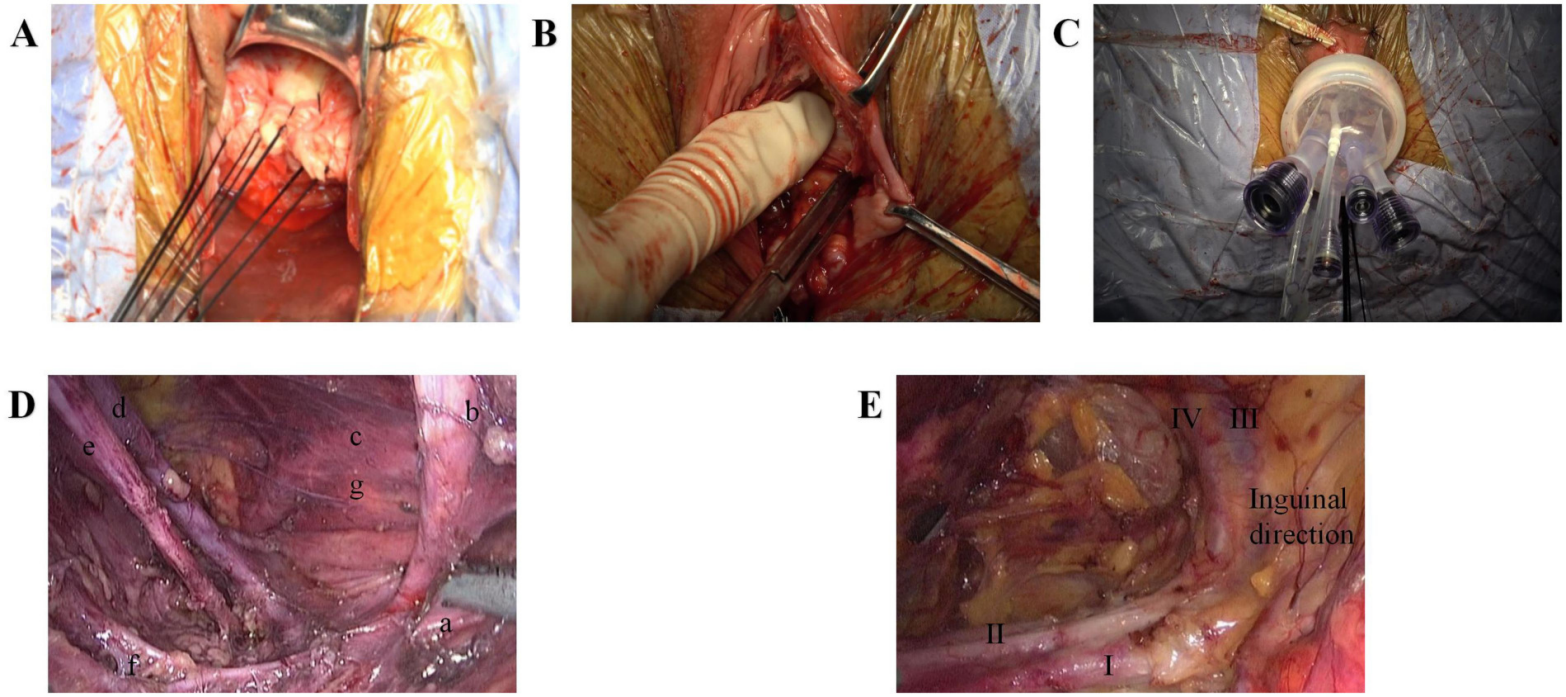
Main intraoperative steps and landmark-based navigation for retropubic extraperitoneal VEEL. **(A)** Tumour-concealing vaginal sleeve/cuff. **(B)** Development of the retropubic/perivesical space behind the descending pubic ramus. **(C)** Establishment of the extraperitoneal working cavity with a transvaginal access platform and CO2 insufflation. **(D)** Landmark-based orientation in the obturator/external iliac region: uterine artery (a), lateral umbilical ligament (b), right ovarian artery (c), right external iliac vein (d), right obturator nerve (e), right internal iliac artery branch (f), and right ureter (g) **(E)** Retropubic view toward the inguinal direction highlighting the external iliac vein (I), external iliac artery (II), inferior epigastric artery (III), and inferior epigastric vein (IV).

**Figure 2:**
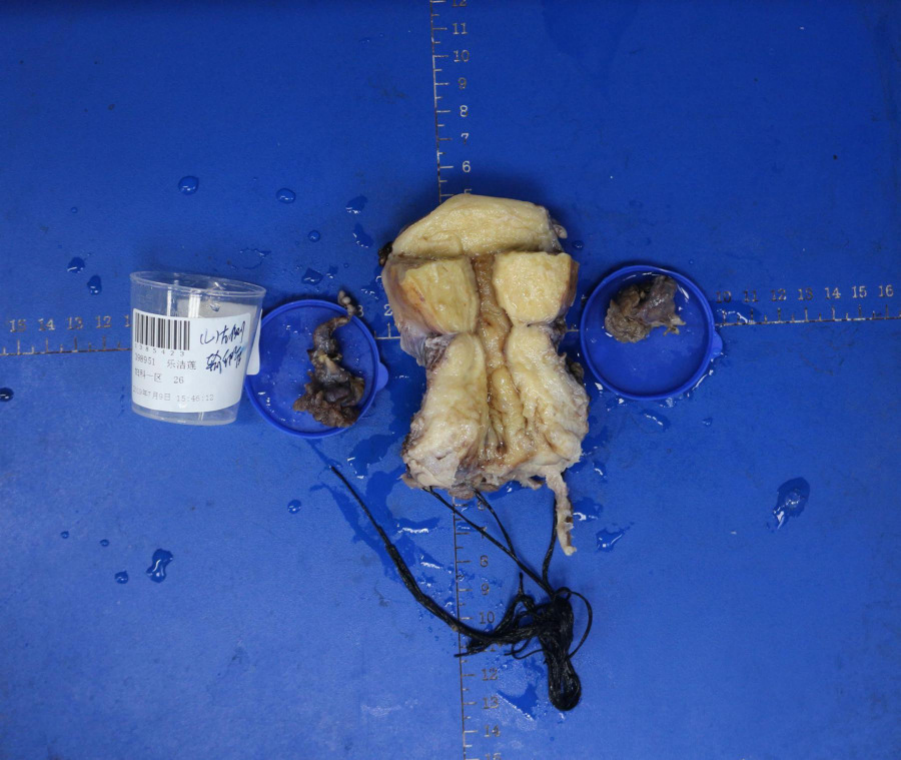
Representative gross surgical specimen following radical vaginal surgery (image from one case). A high-resolution version will be provided in the revised submission.

Mechanical ureteral catheters were used to facilitate ureteral identification in this early experience; as a modern refinement, intra-ureteral indocyanine green (ICG) instillation with near-infrared fluorescence may allow touch-free ureteral visualization and will be considered in future cases. Sentinel lymph node (SLN) biopsy was performed in two patients (patients 3 and 5) via the extraperitoneal transvaginal route, but fluorescence-guided SLN mapping and intraoperative frozen section were not routinely applied in this early experience; all nodal specimens underwent final histopathology (Figures 1, 2).

## 6 Follow-up and outcomes

All five procedures were completed transvaginally without conversion to laparotomy or placement of abdominal trocars. No intraoperative complications were recorded. The mean operative time for the VEEL component was 147.4 min (range 100–182), and mean estimated blood loss was 94 mL (range 50–150).

Pelvic lymph node yield was 26, 21, 14, 34 and 10 nodes per patient, respectively, and all nodes were negative for macrometastases on final pathology. Micrometastases and isolated tumour cells were not specifically assessed in this series. Postoperative bowel function returned by postoperative day 2 in all patients. The most frequent postoperative morbidity was pelvic lymphocele/collections (2/5). One symptomatic lymphocele required laparoscopic drainage after unsuccessful percutaneous aspiration (Clavien–Dindo grade IIIb), and one was detected on ultrasound and managed conservatively.

Final pathological staging remained unchanged in four patients; one patient was upstaged to FIGO IIA1 on final pathology due to vaginal involvement. To improve oncologic transparency, we report patient-level pathological risk factors guiding adjuvant therapy, including tumour size, depth of stromal invasion, lymphovascular space invasion (LVSI), margin status, and nodal assessment (Table 3). Two patients received adjuvant therapy; the patient-level indications and relevant intermediate-/high-risk criteria frameworks (Sedlis and Peters) are summarised in Table 3.

**Table 3:** Final pathology and risk factors guiding adjuvant therapy.

Patient	Histology	Tumor size (mm)	Stromal invasion (mm/fraction)	LVSI	Margins	LN status (macro/micro/ITC)	Adjuvant therapy & criterion
1	AC (AIS + microinv.)	10.0 × 5.5 × 4.0cm	<3/microinv.	+	Vaginal margin (-)	0/26; micro/ITC NR	No
2	SCC (non-ker.); IIA1 (vag. inv.)	10.5 × 5.0 × 3.5cm	≥5/>1/2	+	Parametrium (-); cone margin (+); VaIN3 margin (+); ca margin NR	0/21; micro/ITC NR	CCRT; IIA1 + deep inv. (>1/2) + LVSI (+)
3	SCC (residual post-cone)	3.5 × 3.0 × 2.0cm	4/sup 1/3	–	Endo/ecto margins (-)	0/14; micro/ITC NR	No
4	SCC (non-ker.)	7.0 × 5.0 × 2.5cm	4/mid 1/3	+	Parametrium (-); vaginal margin (-)	0/34; micro/ITC NR	CT; indication NR
5	SCC (microinv.); no residual in completion specimen	6.5 × 1.5 × 0.5cm	4/mid 1/3	±	Cone margin (-); no residual Ca/SIL	0/10; micro/ITC NR	No

AC, adenocarcinoma; AIS, adenocarcinoma *in situ*; CCRT, concurrent chemoradiotherapy; CT, chemotherapy; ITC, isolated tumour cells; LVSI, lymphovascular space invasion; N/A, not applicable; NR, not reported; SCC, squamous cell carcinoma; VaIN, vaginal intraepithelial neoplasia.

## 7 Discussion

This report describes a fully transvaginal radical strategy for selected patients with early-stage cervical cancer. The specific technical nuance is the use of a retropubic extraperitoneal corridor created behind the descending pubic ramus to perform vaginal endoscopic extraperitoneal lymphadenectomy (VEEL) without intentional peritoneal entry, followed by radical vaginal hysterectomy or trachelectomy. Prior work by Baekelandt and others has described retroperitoneal vNOTES access for sentinel node biopsy and, more recently, vNOTES radical hysterectomy (3–7). Our contribution is therefore not the concept of retroperitoneal vNOTES itself, but rather the retropubic access route and its integration with Schauta-type radical vaginal surgery.

From an oncologic standpoint, minimally invasive radical hysterectomy has been questioned after reports of inferior survival in some settings (9, 10), prompting renewed emphasis on tumour containment and minimal tumour handling. Our workflow incorporates early circumferential vaginal sleeve/cuff creation, avoids a uterine manipulator, and aligns with no-look/no-touch concepts intended to reduce tumour disruption.

Several technical points may contribute to procedural safety when operating from an unfamiliar endoscopic perspective. First, standardisation was prioritised through a stepwise, landmark-guided sequence to reduce cognitive load and variability. Second, early identification of stable landmarks (inferior epigastric vessels, external iliac vein, obturator nerve, ureter) supports continuous orientation and may mitigate the risk of vascular or ureteral injury. Third, radical resection was performed vaginally using established anatomic principles and terminology consistent with modern radical hysterectomy classifications (11).

Pelvic lymphocele/collections were observed in 2/5 patients (including one Clavien–Dindo grade IIIb case), consistent with the extraperitoneal setting where lymphatic fluid is less readily absorbed by the peritoneum (10). This experience highlights the need for protocolised prevention and early detection. Potential preventive measures include meticulous sealing or clipping of lymphatic channels, avoidance of unnecessary skeletonisation of major vessels, selective use of pelvic drains, and consideration of peritoneal fenestration when a peritoneal breach occurs. Future iterations of this approach should prospectively track lymphatic outcomes and incorporate evidence-based lymphocele-prevention strategies from gynecologic oncology practice (12, 13).

Sentinel lymph node (SLN) mapping is increasingly recommended for appropriately selected early-stage cervical cancer to reduce lymphatic morbidity while maintaining staging accuracy (1). Feasibility of retroperitoneal vNOTES SLN biopsy has been reported since 2022, including a preliminary series in endometrial cancer and a cervical cancer case report (4, 5). In our early experience, three patients underwent systematic pelvic lymphadenectomy for nodal assessment and two underwent extraperitoneal SLN biopsy; however, fluorescence-guided SLN mapping with ICG and standardized ultrastaging for micrometastasis/isolated tumour cells were not uniformly implemented. In light of the observed lymphocele burden, we now explicitly acknowledge this as a key limitation and will prioritize guideline-concordant SLN mapping (with ICG and appropriate pathological assessment) in future patients with IA2–IB1 disease where applicable.

Patient selection is likely to be pivotal for both feasibility and safety. Based on our experience, suitable candidates include those with small-volume FIGO 2018 IA2–IB1 disease, without bulky or markedly endophytic tumours, and with adequate vaginal access. Prior conisation or an exocervical lesion may facilitate early containment. Conversely, extensive prior pelvic surgery, suspected severe parametrial fibrosis, or limited vaginal exposure may compromise extraperitoneal workspace creation and safe dissection. These considerations should be evaluated within a multidisciplinary tumour board and aligned with contemporary guideline recommendations (1).

This case series is limited by its small sample size, single-centre design, absence of a comparator group, and performance by a highly specialised team, which may restrict generalisability. Although follow-up ranged from 6 to 64 months, larger prospective cohorts are required to confirm long-term oncologic safety and functional outcomes. In addition, lymphatic complications deserve focused optimisation, and future work should include prospective multi-centre evaluation with predefined endpoints (including SLN mapping uptake, lymphocele incidence, and patient-reported outcomes).

## 8 Patient perspective

Formal qualitative interviews were not conducted. During routine follow-up, patients reported satisfaction with rapid postoperative recovery and the absence of abdominal scars. None reported bowel, bladder or sexual function concerns within the follow-up period described.

## Data Availability

The raw data supporting the conclusions of this article will be made available by the authors, without undue reservation.
